# Study on Spatial Distribution Equilibrium of Elderly Care Facilities in Downtown Shanghai

**DOI:** 10.3390/ijerph19137929

**Published:** 2022-06-28

**Authors:** Xiaoran Huang, Pixin Gong, Marcus White

**Affiliations:** 1 School of Architecture and Art, North China University of Technology, Beijing 100144, China; gongpixinncut@mail.ncut.edu.cn; 2 Centre for Design Innovation, Swinburne University of Technology, Hawthorn, VIC 3122, Australia; marcuswhite@swin.edu.au

**Keywords:** elderly care facilities, 2SFCA, spatial accessibility, autocorrelation analysis

## Abstract

With the growing challenge of aging populations around the world, the study of the care services for older adults is an essential initiative to accommodate the particular needs of the disadvantaged communities and promote social equity. Based on open-source data and the geographic information system (GIS), this paper quantifies and visualizes the imbalance in the spatial distribution of elderly care facilities in 14,578 neighborhoods in downtown (seven districts) Shanghai, China. Eight types of elderly care facilities were obtained from Shanghai elderly care service platform, divided into two categories according to their service scale. With the introduction of the improved Gaussian 2-step floating catchment area method, the accessibility of two category facilities was calculated. Through the global autocorrelation analysis, it is found that the accessibility of elderly care facilities has the characteristics of spatial agglomeration. Local autocorrelation analysis indicates the cold and hot spots in the accessibility agglomeration state of the two types of facilities, by which we summarized the characteristics of their spatial heterogeneity. It is found that for Category−I, there is a large range of hot spots in Huangpu District. For Category−II, the hot-spot and cold-spot areas show staggered distribution, and the two categories of hot spot distribution show a negative correlation. We conclude that the two categories are not evenly distributed in the urban area, which will lead to the low efficiency of resource allocation of elderly care facilities and have a negative impact on social fairness. This research offers a systematic method to study urban access to care services for older adults as well as a new perspective on improving social fairness.

## 1. Introduction

### 1.1. Background

In the years to come, China’s population of older adults is expected to continue developing rapidly [1]. Data from the National Bureau of statistics shows that by 2021, China’s population over the age of 65 accounted for 14.20% of its population [2]. According to the WHO criteria for aging, having more than 14% of the population aged 65 indicates that China has entered an “aged society”. At the same time, as the group commonly referred to as “post-1960s baby boomers [3,4]” will be aging in the next 5–10 years, China is expected to enter a “super-aged society” around 2033 with more than 20% of its population being 65 or above [5]. The emergence of the Chinese ”baby boomers” was a few years later than its counterpart in the West, but it was sustained longer and had a more significant impact on the birth rate [6]. After the natural disasters in 1962, the national economy had gradually recovered by the 1970s along with a strong compensatory fertility momentum, resulting in the largest population growth in Chinese history [7]. Subsequently, the elderly dependency rate (defined as the ratio between the elderly population and the working-age population [8]) will rise radically in the next decade, thereby putting strain on the government and society to allocate resources for healthcare, rehabilitation, and old-age care [9,10]. Facing these evolving ageing challenges, local governments have initiated proactive policies successively, such as the goal of “9073” (Shanghai, Jiangsu, Sichuan and Guangdong) and “9064” (Beijing), which have eventually merged a basic pension system for older adults of “home-based, community-depended and institution-supported” [11,12], committed to the collaborative cooperation of family, community, and institutions. Since 2012, China’s old-age care industry has ushered in a period of rapid development, and various elderly care facilities have emerged in first-tier cities [13]. However, it is shown that there is an obvious mismatch between supply and demand in the distribution of pension resources, and even the current facilities in some well-developed areas have not yet met the planning requirements [14,15,16,17,18].

Due to the decline of physical function, the life of the elderly is more likely to be challenged and their adaptability to the environment is reduced [19,20,21,22]. Therefore, the elderly who have easier access to pension facilities and resources can get timely care for their bodies and meet their basic living needs with less transportation cost, which is conducive to prolonging their life span and improving their happiness [23,24]. Access refers to the possibility of reaching a place, goods, services, etc., also known as access opportunity and convenience [25]. Accessibility assessment is one of the main methods of research on the rationality of the spatial distribution [26,27,28], which is widely used in the layout evaluation of public service facilities, such as education, medical treatment, elderly care facilities, parks, and green spaces [29,30]. Countries that also face serious aging problems, such as Japan [31,32], South Korea [33], Finland [34], and the United States [35,36], have also attracted extensive attention from scholars to study the accessibility of public service facilities. With the development of new urban science, multi-source data provide important support for evaluating the accessibility of elderly care facilities [37], such as POI (point of interest) [38,39,40], street-view images [41,42,43,44], smart cards [45], and mobile phones [46]. Compared with the traditional small-scale sampling method, the use of multi-source data can achieve large-scale and rapid collection of the objective distribution of urban pension service resources [47]. Methods to study accessibility usually include the gravity model method, huff model, kernel density method, network analysis method, and 2-Step floating catchment area (2SFCA) method [35,48,49], among which the 2SFCA is widely favored by researchers. The 2SFCA method stems from the floating catchment area (FCA) approach, which uses spatial interaction processes in the manipulation of supply and demand [50]. Huang et al. used the 2SFCA method to measure spatial access to health care for older adults [51]. He et al. [52], using the 2SFCA method and potential model to measure spatial accessibility, respectively, compare the two methods in different types of elderly care facilities. Fan et al. [53,54] used the Gaussian two-step floating catchment area (G2SFCA) method to assess the accessibility to community-based services. Han and Luo [55], based on multi-source data and from the perspective of supply–demand matching, applied the improved 2SFCA method to evaluate the accessibility of home-based elderly care facilities. Li and Liang [56] applied the enhanced 2SFCA algorithm to study the spatial balance of community-based elderly care facilities. Cheng et al. examined the walking accessibility to recreational amenities for older adults, with an explicit focus on equity, and presented the distribution of accessibility across zones [57].

Previous studies have the following limitations. Firstly, the research units are often too large (mainly at the district level or sub-district level) and scarcely consider the cost of travel time and living range, resulting in the research conclusions mismatching the everyday reality of older adults. Secondly, previous studies mostly considered the layout equilibrium of a single type of elderly care facility. In fact, multiple types of resources should be considered to explore the accessibility of elderly care facilities; especially, at the community level, older adults could obtain better service resources at a lower transportation cost. Thirdly, global conclusions cannot adequately explain the spatial heterogeneity and achieve accurate positioning of areas with uneven distribution of resources.

### 1.2. Framework

**Figure 1:**
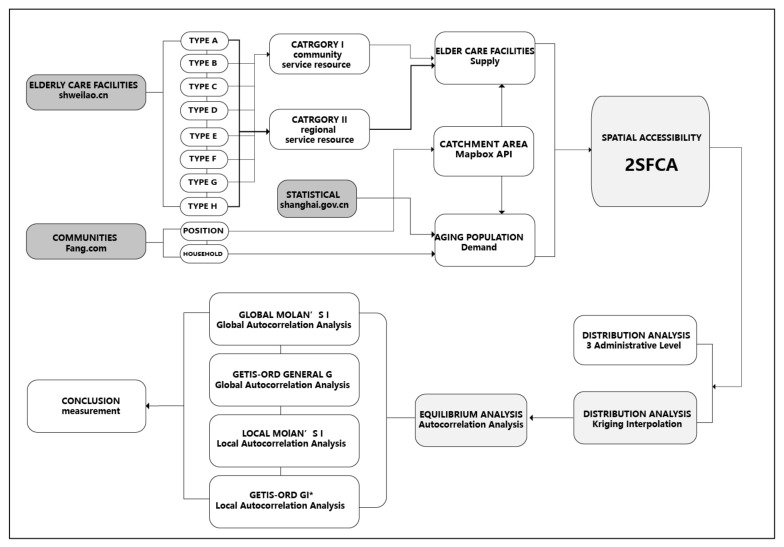
Research framework.

Previous studies on accessibility, with the use of 2SFCA method, mostly set the catchment area as the travel range within a travel time of 0.5–1.5 h [58,59,60]. However, its limited spatial scope impacts the actual accessibility results, covering up the imbalance of accessibility in reality. There are two aspects to consider for community residents: first, older adults living at home can easily access elderly care facilities; secondly, children can easily visit their parents living in elderly care facilities. The 15-min community-life circle is the basic unit for community life in Shanghai. That is, within the range of 15-min walking cost, essential services and public spaces should be provided to form a safe, friendly, and comfortable social living environment [61]. In recent years, the principle of community-life circle issued by the government has set the scope of supply and demand services of public facilities [62]. For example, “Building a diversified and integrated 15-min community life circle” is proposed in the Shanghai Master Plan 2017–2035 [63] and Shanghai Planning Guidance of 15-Minute Community-Life Circle [61]. The goal is to reach about 99% of the coverage of community service facilities in Shanghai within 15 min by 2035. In February 2022, Shanghai Civil Affairs Bureau issued the draft of Planning for the Layout of Shanghai Elderly Care Facilities (2021–2035), which proposed to ensure the full coverage of a 15-min community-life circle of basic care services for older adults and improve the serviceability for key aging groups such as living alone, advanced age, disability, and dementia [64]. Therefore, this paper employed the 15-min community-life circles as the study units. The proposed research framework of this paper is shown in Figure 1.

Based on the findings of previous studies, this paper improves the 2SFCA method to calculate the accessibility of elderly care facilities for each community, based on the resources of elderly care facilities and the needs of the aging population. The data of elderly care facilities, aging population, and community-life circle were obtained from the website using python. Then, the improved 2SFCA method was adopted, from the supply and demand side, respectively, to calculate the accessibility of elderly care facilities. The calculation results were visualized as accessibility distribution maps of care resources for older adults in ArcGIS, and three-level comparisons (community, sub-district, and district) were conducted considering administrative region matching and Kriging interpolation analysis without administrative region matching. Finally, we used spatial autocorrelation analysis to investigate the characteristics of spatial agglomeration.

Compared to the accessibility research focus on the administrative region boundary, the study of resource accessibility in the community-life circle of older adults has more practical significance. Moreover, paying attention to the distribution of pension resources is to resolve not only the current pragmatic needs of older adults but also an important measure to improve social equity, so as to avoid insufficient resources or vacant waste due to uneven spatial distribution. This research reflects the accessibility distribution characteristics of elderly care facilities in downtown Shanghai and provides an essential reference for the planning agenda revision and the policy-making progress in the near future. The proposed research framework and digital techniques can be applied in other cities and potentially adopted in other disciplines such as epidemiology studies.

## 2. Research Site and Data Acquisition

### 2.1. Research Site

**Figure 2:**
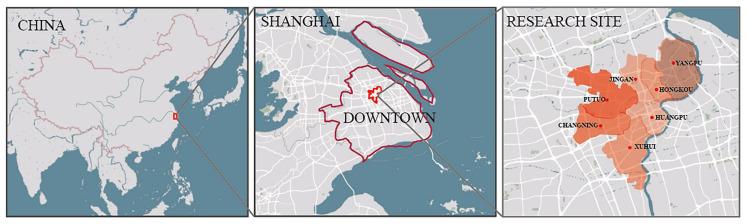
Research site (administrative boundary of Shanghai).

Shanghai is the city with the highest level of urbanization development in China. With the improvement of China’s economic development level and national economic strength, more cities will enter the development stage of Shanghai at this time. Therefore, taking Shanghai as the research site has forward-looking significance. On the other hand, Shanghai has a high degree of urban information construction, with a high degree of information disclosure and easy access to various kinds of information [65,66]. According to the data of Shanghai Statistical Yearbook 2021, Shanghai, the first city entering the aging society in China, has a large population of registered older residents (60 or above years old) of 5,324,100, accounting for 36.08% of the total demographic. The aging problem in downtown Shanghai is even more severe (Huangpu District of 41.7%, Hongkou of 42.5%, Putuo District of 41.1%, Jingan District of 40.1%, Changning District of 39.1%, Yangpu District of 38.8%, and Xuhui District of 35.9%). With the elderly dependency rate increasing year by year, the city has been entering a state of deep aging society [67]. As the population in downtown Shanghai is extremely concentrated, essential service distributions for the highly populated aged community are particularly significant. Therefore, the research scope of this study has focused on the distribution equilibrium of elderly care facilities in the community-life circles in downtown Shanghai. The research site is shown in the Figure 2 below.

### 2.2. Data Acquisition

#### 2.2.1. Acquisition of Community Location and Community Life Circle Data

A python crawler was created to extract data of 27,621 communities (containing location coordinate, household count, average property price, and other information) from the real estate business website (fang.com, accessed on 15 January 2021), which includes 14,578 communities in downtown Shanghai. Then, the location coordinates of the communities were used to link with the Mapbox’s Isochrone API (mapbox.com, accessed on 25 April 2022)to obtain the geographic data of the community-life circle of all communities. The Isochrone API allows us to request polygon features that show areas that are reachable within a specified amount of time from a location. The time for this study was set at 15-min walking time cost in accordance with the Shanghai Master Plan’s current urban development criteria [61]. According to the Shanghai Statistical Yearbook 2021, the average household size [68] and the proportion of the aging population can be attained, which allow us to approximate the number of older adults in each community.

#### 2.2.2. Acquisition of Elderly Care Service Facilities and Service Circle Data

Older adults’ choice of surrounding care services is not limited to the boundaries of administrative regions, and the communities located at the edge of administrative regions may choose care services across administrative boundaries. So, the scope of elderly care facilities obtained in this paper is extended to the surrounding areas of research site. Shanghai elderly care service platform (shweilao.cn, accessed on 20 April 2022) has registered the data of different kinds of elderly care facilities in Shanghai, with various types, complete data, and information authority. Therefore, this study obtains various types of elderly care facilities from this platform. The effective data obtained this time are: nursing home, Type-A (706), providing centralized care services; elderly care home, Type-B (170), providing medium and short-term care services; elderly daycare institution, Type-C (725), providing daycare services; meal aid service point, Type-D (1741), providing catering service for the elderly; community elderly care service organization, Type-E (259), providing door-to-door services; comprehensive services center, Type-F (360); and nursing station, Type-G (168) and nursing hospital, Type-H (54). Since the map coordinates used by this platform are Amap coordinates, coordinate conversion shall be carried out before data cleaning. In order to facilitate the research and take into account the service scope of elderly care facilities, the catchment area is distinguished into 2 categories. As pointed by Yang et al., the catchment size may also vary according to the type of provider and the cost of transportation [69]. Category−I, provide medium and short-term services, whose catchment size is set as a 30-min walking distance (Type-B, -C, -D, -E, -F, and -G); Category−II, provide long-term and comprehensive centralized care services, whose catchment size is set as a 15-min driving distance (Type-A and Type-H). Two categories of catchment area data are obtained from the Mapbox platform.

## 3. Method

### 3.1. Calculating the Spatial Accessibility of Elderly Care Facilities

**Figure 3:**
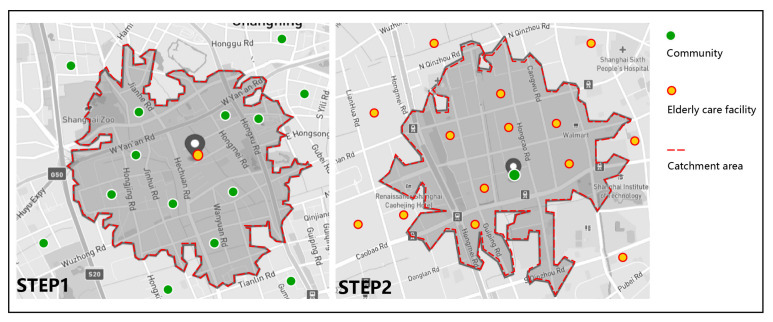
Process diagram of 2SFCA (from left to right: Step 1: calculating the supply/demand ratio; Step 2: calculating the accessibility of elderly care facilities).

The spatial accessibility of facilities is a crucial criterion for assessing the rationality of public service layout [70]. The supply–demand ratio indicates the magnitude and convenience of inhabitants’ access to public service amenities, and it may be used to visualize the spatial distribution of facilities in equilibrium [71]. The 2-step floating catchment area (2SFCA) approach, first proposed by Radke and Mu [72] but later modified by Luo and Wang [50], is a special case of the gravity model. It has most of the advantages of a gravity model and is also intuitive to interpret [58]. This method calculates facility accessibility through two steps [50]: the first step is to calculate the supply–demand ratio, and the second step is to calculate the spatial accessibility of facilities (Figure 3), which can account for the influence of supply scale, demand scale, and spatial impedance factors between supply and demand sites on elderly facility accessibility.

However, traditional 2SFCA has two problems: the unreasonable setting of the catchment area and the homogeneity of weight in the search domain [73], resulting in a certain deviation between the calculation results and the reality. For the effective catchment area, some scholars have explored the variable scope. For example, McGrail and Humphreys [74] set a different search radius according to the population density of regions. Luo and Whippo [75] dynamically determine catchment sizes by incrementally increasing the catchment radius until a base population and a physician-to-population ratio are satisfied. Mao and Nekorchuk [76] develop a modified 2SFCA framework incorporating multi-mode transportation. For the distance decay of weight in the catchment area, some studies have introduced distance decay functions, including Gaussian function [26,77], kernel density function [78], and power function [50], addressing the problem of uniform access within the catchment by applying weights to different travel time zones to account for distance decay [79].

This work calculates the accessibility of elderly care facilities and makes improvements based on the advantages of previous research results. Firstly, the elderly care facilities are classified into two categories: Category−I (Type-B, -C, -D, -E, -F, and -G), which is the community service resource; and Category−II (Type-A and Type-H), which is a regional service resource. The service scope of the two categories is determined based on the time cost. Category−I is set as a 30-min walking range, and Category−II is set as the 15-min driving range. Category−I considers the accessibility of elderly care facility resources for older adults who depend on community and home-based elderly care. Two aspects are considered for Category−II: first, the potential demand of the older adults living at home; second, the accessibility of their children to visit for the elderly living in institutions. Generally speaking, the two categories of facilities comprehensively consider the elderly care needs under the three modes of home-based care, community-based care, and institution-based care, as well as the accessibility requirements for children to visit conveniently.

In the catchment area, the Gaussian equation is used for distance decay to determine the weight of supply and demand capacity of different facilities.

(1) $$G\left(d_{kj},d_{0}\right)=\left\{\begin{array}{l} e^{-\left(1/2\right)\times {\left(d_{kj}/d_{0}\right)}^{2}}-e^{-\left(1/2\right)},\;\;\;\;ifd_{kj}\leq d_{0}\\ 0,\;\;\;\;\;\;\;\;\;\;\;\;\;\;\;\;\;\;\;\;\;\;\;\;\;\;\;\;\;\;\;\;\;\;\;\;\;\;,\;\;\;\;ifd_{kj}>d_{0} \end{array}\right.,$$

where $d_{kj}$ is the distance between the supply point *k* and the demand point *j*, and $d_{0}$ is the threshold.

With the following calculation processes, the 2SFCA method estimates the accessibility of elderly care facilities in two phases, depending on the place of supply and demand, respectively.

Step 1: considering that the choice of care resources for older adults is not limited to the boundaries of administrative regions, the supply–demand ratio is calculated by using the elderly care facilities and the community data in total Shanghai, to meet the actual choice of older adults. Calculate the elderly care facility supply–demand ratio $R_{j}$. $S_{j}$ is the number of facility, *k* is community, and $P_{k}$ is the population of older adults. $d_{0}$ is chosen as the threshold, the range of each catchment area is considered as the walking distance at a 30-min walking time cost for Category−I and 15-min driving time for Category−II, which is shown in in Section 2.2.2, and the total weighted population served is calculated using a Gaussian equation.

(2) $$R_{j}=\frac{S_{j}}{\;\sum_{k\in \left\{d_{kj}\leq d_{0}\right\}}G\left(d_{kj},d_{0}\right)P_{k}},$$

Step 2: calculating the spatial accessibility of each community $A_{i}$. Firstly, the life circle polygon analyzed in Section 2.2.1 was chosen as the catchment area. Using a Gaussian equation, assign weights to the supply–demand ratios $R_{j}$ for each elderly care facility in this spatial scope, and then total these weighted supply–demand ratios to get the accessibility of elderly care facilities for each community.

(3) $$A_{i}=\sum_{l\in \left\{d_{il}\leq d_{0}\right\}}G\left(d_{il},d_{0}\right)R_{j},$$

### 3.2. Calculation on Three Administrative Levels of Spatial Accessibility

With the background that governments at different levels have put forward the construction aim of elderly care facilities, a considerable number of elderly care facilities are proposed to meet the needs of older adults in their administrative region. Therefore, it is crucial to summarize and analyze at different administrative levels. With the use of the ArcGIS platform, the calculation results of accessibility are averaged in three levels: community, sub-district, and district. Then, the accessibility of the three levels is analyzed to find the specific areas with uneven resource distribution.

In fact, the choice of elderly care services is not necessarily limited to administrative region matching. The spatial interpolation method can convert the measured data of discrete points into continuous data surfaces for comparison with the distribution patterns of other spatial phenomena, without considering the administrative boundary [80]. In this paper, the Kriging interpolation method is used to draw isosurface from the community scale.

(4) $${\tilde{z}}_{0}=\sum_{i=1}^{n}\lambda_{i}z_{i},$$

where ${\tilde{z}}_{0}$ is the estimated value at point $\left(x_{0},y_{0}\right)$, $z_{0}=z(x_{0},y_{0}$), and $\lambda_{i}$ is the weight coefficient.

### 3.3. Analysis on Spatial Distribution Equilibrium

In the aspect of spatial distribution research, nearest neighbor hierarchical clustering [81], Ripley’s K function [82], Gini coefficient [16], Shannon entropy [45], and spatial autocorrelation [26,83,84] are often used to explain the aggregation status of facilities in geospatial space. Spatial autocorrelation could observe the correlation between variables close to each other on a spatial scale, more conveniently identifying the areas with a significant imbalance between supply and demand.

In order to analyze the spatial distribution characteristics of elderly care facilities, this paper uses spatial autocorrelation tools to study the resource distribution. Spatial autocorrelation can be used to detect three spatial data distribution modes—clustering, dispersed, and random—which can be divided into global autocorrelation (Global Moran’s I, Getis-Ord General G) and local autocorrelation (local indicator of spatial autocorrelation (LISA), Getis-Ord Gi*tool). Global Moran’s I generally reflects the spatial autocorrelation of the study area, which is used to judge whether there is spatial autocorrelation in the whole, and the Getis-Ord General G method was used to preliminarily judge the agglomeration type [85].

The Global Moran’s I method of global spatial autocorrelation is given as

(5) $$I=\frac{n\sum_{i=1}^{n}\sum_{j=1}^{n}W_{ij}\left(x_{i}-\bar{x}\right)\left(x_{j}-\bar{x}\right)}{\sum_{i=1}^{n}\sum_{j=1}^{n}W_{ij}\sum_{i=1}^{n}{\left(x_{i}-\bar{x}\right)}^{2}},$$

The Getis-Ord General G method of global spatial autocorrelation is given as

(6) $$G=\frac{\sum_{i=1}^{n}\sum_{j=1}^{n}w_{i,j}x_{i}x_{j}}{\sum_{i=1}^{n}\sum_{j=1}^{n}x_{i}x_{j}},\;j\neq i$$

where $x_{i}$ and $x_{j}$ are attribute values for features i and j; $w_{i,j}$ is the spatial weight between feature i and feature j; *n* is the number of features in the dataset; and j≠i indicates that feature i and feature j cannot be same.

The global autocorrelation statistic indicates the presence of clusters, whereas local autocorrelation indicates the location of clusters and the type of spatial association. To further investigate the distribution patterns of geographic accessibility scores, local autocorrelation analysis was used to identify local clusters of accessibility. Due to the characteristics of spatial heterogeneity, there will be different aggregation states in different geographical locations. LISA [86] is suited to study the heterogeneity characteristics of the accessibility agglomeration of elderly care facilities. The Getis-Ord Gi*tool was employed to conduct hot and spot analysis, which can analyze the distribution area of cold and hot spots of the accessibility.

The LISA method of local spatial autocorrelation is given as

(7) $$I_{i}=\frac{n\left(x_{i}-\bar{x}\right)\sum_{i=1}^{n}\sum_{j=1}^{n}w_{i,j}\left(x_{i}-\bar{x}\right)}{\sum_{i=1}^{n}{\left(x_{i}-\bar{x}\right)}^{2}},$$

The Getis-Ord Gi* method of local spatial autocorrelation is given as

(8) $$G_{i}^{*}=\frac{\sum_{j=1}^{n}W_{i,j}x_{j}-\bar{X}\sum_{j=1}^{n}w_{i,j}}{S\sqrt[{2}]{\frac{\left[n\sum_{j=1}^{n}w_{i,j}^{2}-{\left(\sum_{j=1}^{n}W_{i,j}\right)}^{2}\right]}{n-1}}},$$

where $x_{i}$ and $x_{j}$ are attribute values for features i and j; $w_{i,j}$ is the spatial weight between feature i and feature j; and *n* is the number of features in the dataset. When the Gi* statistic is higher than the mathematical expectation and passes the hypothesis test, it is a hot spot; otherwise, it is a cold spot.

## 4. Results and Discussion

### 4.1. The Results of Elderly Care Facilities Density

**Figure 4:**
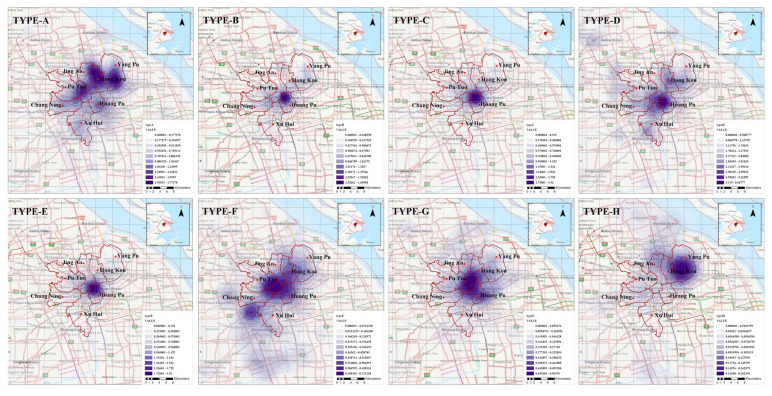
Kernel density diagram of eight types elderly care facilities (Type-A, -B, -C, -D, -E, -F, -G, and -H).

The kernel density diagram of eight types of elderly care facilities is shown in Figure 4; it can find the spatial distribution imbalance in quantity. The distribution of Type-A shows a multi-point agglomeration state and others show single-point agglomeration distribution. Type-A is the nursing home providing long-term care. It has a high degree of aggregation in the north of downtown Shanghai, with YangPu, HongKou, and PuTuo presenting the aggregation points, while several districts in the south have a low degree of aggregation. Type-B, -C, -D, and -E are community-based services, which are mainly concentrated in HuangPu and less distributed in other districts. Type-F and Type-G are mainly concentrated in the border area of PuTuo, JingAn, and HuangPu. Type-H, the nursing hospital, is mainly distributed in YangPu District. From the resource density distribution of the above eight elderly care facilities, the spatial distribution is uneven, and most show the single center’s agglomeration state. As a result, there is often a waste of resources in high-density areas and a shortage of resources in low-density areas. Although kernel density can show the quantity density of various facilities in space, it can not reflect the real relationship between supply and demand and the availability of pension resources for older adults.

### 4.2. The Results of Statistical Analysis on Administrative Regions

**Figure 5:**
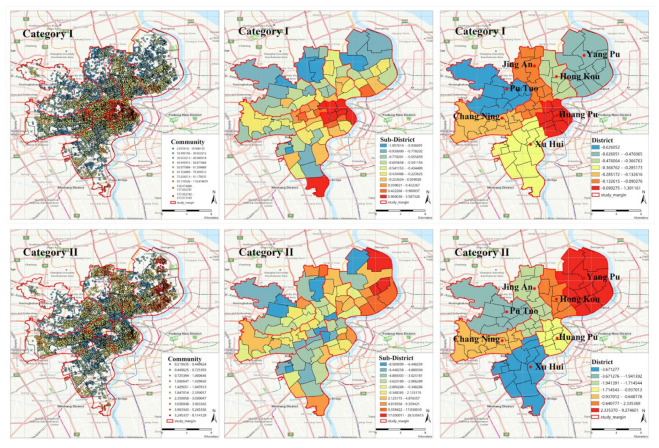
Statistical analysis of administrative regions (community, sub-district, and district).

The distribution trend of the accessibility of elderly care facilities in downtown Shanghai is shown in Figure 5. Considering the matching of administrative regions, the research is carried out at the community, sub-district, and district levels, respectively. For Category−I, the high accessibility value at the community level is mainly distributed in the central area of HuangPu District, extending outward in strips. At the sub-district level, it can be seen that the accessibility of seven sub-districts in the north of HuangPu District is the best, and the other Huajing town of Xuhui District also has a high value. At the county level, Huangpu District is the best and PuTuo District is the lowest. It is obvious that the data at the district level has erased the characteristics of internal diversity. Generally speaking, the high accessibility of social resources of Category−I is too concentrated in the communities in the north of Huangpu District. For Category−II, at the community level, the distribution of accessibility values is relatively balanced. At the sub-district level, most sub-districts in Yangpu District have higher accessibility. At the district level, YangPu District has the best accessibility and XuHui has the worst accessibility. The global spatial distribution of Category−II is relatively even.

**Figure 6:**
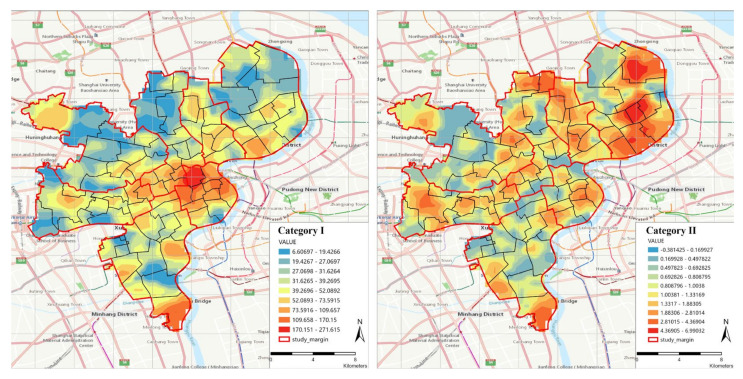
Kriging interpolation of two categories (without considering administrative region matching).

From the Kriging interpolation results (Figure 6), it can be seen that the perspective of administrative region matching cannot show the actual accessibility distribution, covering up the imbalance of facts. For example, for Category−I, the value of HuangPu District is high both at the sub-district and district level, but, most importantly, the highest value appears in the Nanjing East Road sub-district, which radiates outward with it as the center. Without considering the matching of administrative regions, as for the other six sub-districts (which have the same value as Nanjing East Road), there will be no obvious advantage compared to other regions. Moreover, more real features will be obscured at the district level. For Category−II, taking Yangpu District as an example, the high value from the perspective of the sub-district and district level has covered the low-value phenomenon in some areas in the East Yangpu District. If the distance to the elder care facility is long, people could consider moving to a location where the facility is situated nearer [87], which will aggravate the run-on pension resources and cause a greater waste of resources.

Without considering the matching of administrative regions, this result is closer to the real value, and the government should consider the actual distribution when assessing which region meets the standard.

### 4.3. The Results of Global Autocorrelation Analysis

**Figure 7:**
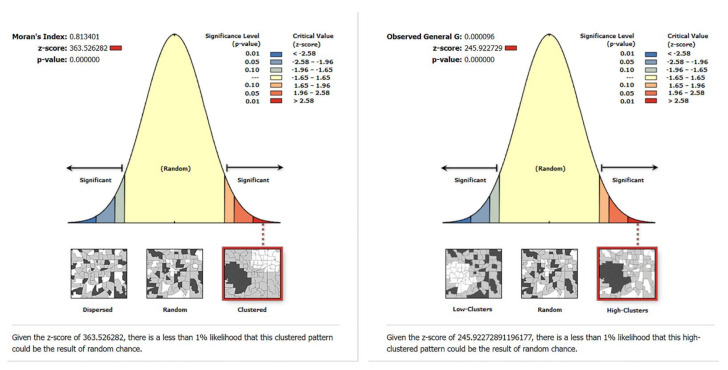
Global Moran’s I (Category−I); Getis-Ord General G (Category−I).

For Category−I, with the Global Moran’s I analysis, the *p*-value is less than 0.00001 and the Z-score is 363.526282, which is highly significant, indicating that the elderly care facilities of Category−I are clustered (Figure 7). High and low-value clustering (Getis-Ord General G) shows that the *p*-value is less than 0.00001 and the Z-score is 245.922729, indicating that high-value clustering occurs in facility accessibility.

**Figure 8:**
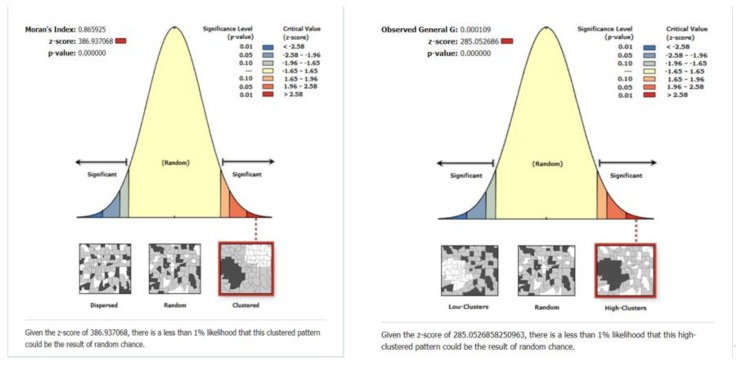
Global Moran’s I (Category−II); Getis-Ord General G (Category−II).

For Category–II, the Global Moran’s I analysis (*p*-value < 0.00001, Z-score = 386.937068) and Getis-Ord General G (*p*-value < 0.00001, Z-score = 285.052686) results indicate that the null hypothesis is denied, and show high-value agglomeration (Figure 8). From the global correlation analysis, it can be seen that both categories of elderly care facilities have spatial agglomeration, and the spatial agglomeration presents high-value clustering.

### 4.4. The Results of Local Autocorrelation Analysis

**Figure 9:**
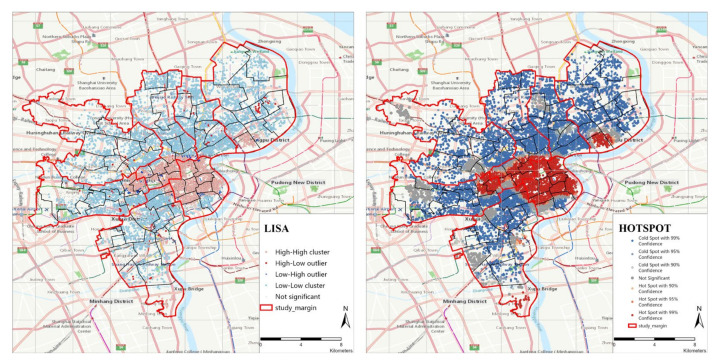
Local autocorrelation analysis of Category−I (LISA, Getis-Ord Gi*).

As shown in Figure 9, the figure LISA of Category−I shows high–high aggregation in the central area of downtown (mainly Huangpu District) and low–low distribution in the surrounding urban areas. As seen from the cold and hot spots map (Getis-Ord Gi*), the hot spots are mainly concentrated in some sub-districts of Huangpu District and Jing’An District. It can be concluded that the service distribution of Category−I is uneven in the central urban area of Shanghai. Category−I is more closely related to home-based and community-based elderly care facilities. Older adults may also be more limited in their choice of health care providers than younger adults, due to the increased likelihood of cushioning from intrinsic and extrinsic mobility constraints such as physical disabilities [21,88]. Older adults might require more social and health services and have more available time to carry out their out-of-home activities. Limited access to primary care may lead to higher levels of chronic disease, the consumption of more medication, and shorter life expectancy [89]. At present, the elderly living at home occupy the main part of the care pattern. Communities with abundant elderly care service resources can effectively help the elderly spend their old age in peace and reduce the transportation costs and economic costs of medical care in other places. For the government, improving resource allocation and accessibility can effectively help older adults to enjoy their time. Moreover, a more convenient community-life circle environment can effectively ease the current shortage of institutional nursing beds.

**Figure 10:**
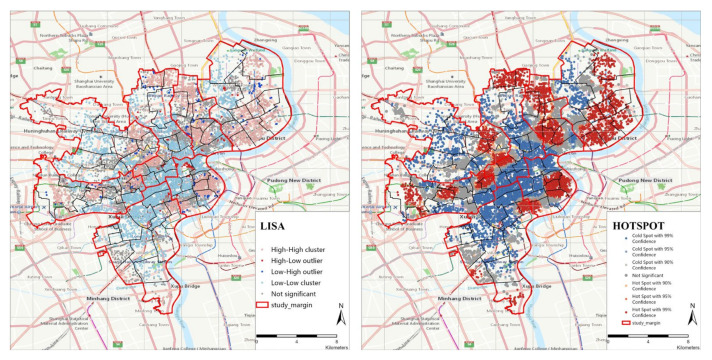
Local autocorrelation analysis of Category−II (LISA, Getis-Ord Gi*).

The investigation results are shown in Figure 10. The analysis of the two methods has obtained similar results in space. The figure LISA of Category−II shows a high–high aggregation phenomenon and presents multipoint distribution. It can be seen from the cold- and hot-spot map (Getis-Ord Gi*) that the hot spot areas occupy a wide range in HongKou District and YangPu District, while the Xuhui District and Jing’An District are mainly occupied by the cold-spot area. It is worth noting that hot spots are more likely to appear in the marginal areas of each district, the reason for this is that the distribution of elderly care facilities may be affected by the land price. Compared with Category−I, we found that some high-value areas of Category−I become cold spots in Category−II, such as several sub-districts bordering Huangpu District and Jing’an District. Generally speaking, the distribution of Category−II shows a multi-point and relatively balanced distribution. Since the service range is set to 15-min driving cost (within 15 km) when calculating these facilities, if the service range is expanded, the hot spot area will have a larger range than now.

## 5. Conclusions

With the growing challenge of aging populations around the world, the study of elderly care facilities is an essential initiative to accommodate the particular needs of these disadvantaged communities and promote social equity. Thus, 14,578 communities were selected in this paper, and the accessibility of elderly care facilities in community-life circles was researched, with a focus on the imbalanced distribution in downtown Shanghai. Eight types of elderly care facilities were obtained from the Shanghai elderly care service platform, which were divided into two categories according to their service scale.

This paper comprehensively considers the elderly care needs under the three modes of home care, community care, and institutional care, as well as the accessibility requirements for children to visit. Category−I considers the accessibility of elderly care facilities resources for older adults who depend on community and home-based elderly care. Two aspects are considered for Category−II: first, the potential demand for Category−II by the older adults living at home; second, for the elderly in institutions, the accessibility for their children to visit conveniently.

The kernel density analysis of the aggregation state of all kinds of facilities indicates that the spatial distribution density of various facilities is extremely uneven, and the density in Huangpu District is the highest. The improved Gaussian 2-step floating catchment area method was used to calculate the accessibility of two category facilities. Considering the matching of administrative regions, the visual description and comparative analysis are carried out at the three administrative levels: community, sub-district, and district. It is found that for Category−I, the accessibility of residential areas in HuangPu District is the best, and for Category−II, YangPu District is the best. Then, without considering matching administrative regions, we pay attention to the agglomeration state of community-level accessibility. From the results of Kriging interpolation, we found that some real features were covered because of matching administrative regions. Through the global autocorrelation analysis, we found that the accessibility of two categories has a high-value agglomeration state in space. Then, the autocorrelation analysis was used to analyze the spatial heterogeneity. It was found that for Category−I, there was an extensive range of hot spots in Huangpu District. For Category−II, the hot-spot and cold-spot areas showed staggered distribution, and the two categories of hot spot distribution showed a negative correlation. In general, it was concluded that two categories are not evenly distributed in the urban area, which will lead to the low-efficiency resource allocation of elderly care facilities and have a negative impact on social pensions for older adults.

Admittedly, there are still some limitations to this paper. In terms of data acquisition accuracy, the community-level aging population data can only be obtained through a prediction method, which may cause deviations compared to the actual situation. For the setting of the service scope of elderly care facilities, although referring to previous studies, there may be a more appropriate numerical settings based on systematic sensitivity analysis. However, due to the wide research scope of this paper, the comparative study of this paper has proven to be adequate in revealing the problems of resource distribution. In brief, the research conclusions of this paper can identify areas with insufficient resource distribution for the future layout optimization of elderly care facilities in Shanghai. The proposed framework can provide insights for planning schemes in other similar cities, whereas the workflow of this project could also potentially offer a reference for the research of other public facilities.

## Data Availability

fang.com (accessed on 15 January 2021) for the data of communities (containing location coordinate, household count, average property price, and other information); shweilao.cn (accessed on 20 April 2022) for the data of elderly care facilities; tjj.sh.gov.cn (accessed on 20 April 2022) for the statistical data; and mapbox.com (accessed on 25 April 2022) for the data of geographic data of the community-life circle and service circle of elderly care facilities.
